# Serum Metabolomics Study Based on LC-MS and Antihypertensive Effect of* Uncaria* on Spontaneously Hypertensive Rats

**DOI:** 10.1155/2018/9281946

**Published:** 2018-04-04

**Authors:** Ana Liu, Yan-Jun Chu, Xiaoming Wang, Ruixue Yu, Haiqiang Jiang, Yunlun Li, Honglei Zhou, Li-Li Gong, Wen-Qing Yang, Jianqing Ju

**Affiliations:** ^1^Pharmacy School, Shandong University of Traditional Chinese Medicine, Jinan 250355, China; ^2^Traditional Chinese Medicine Clinical Research Base for Hypertension of Affiliated Hospital, Shandong University of Traditional Chinese Medicine, Jinan 250011, China; ^3^Experimental Center, Shandong University of Traditional Chinese Medicine, Jinan 250355, China

## Abstract

Our previous studies have shown that* Uncaria* has an important role in lowering blood pressure, but its intervention mechanism has not been clarified completely in the metabolic level. Therefore, in this study, a combination method of HPLC-TOF/MS-based metabolomics and multivariate statistical analyses was employed to explore the mechanism and evaluate the antihypertensive effect of* Uncaria*. Serum samples were analyzed and identified by HPLC-TOF/MS, while the acquired data was further processed by partial least squares discriminant analysis (PLS-DA) and orthogonal partial least squares discriminant analysis (OPLS-DA) to discover the perturbed metabolites. A clear cluster among the different groups was obtained, and 7 significantly changed potential biomarkers were screened out. These biomarkers were mainly associated with lipid metabolism (dihydroceramide, ceramide, PC, LysoPC, and TXA2) and vitamin and amino acids metabolism (nicotinamide riboside, 5-HTP). The result indicated that* Uncaria* could decrease the blood pressure effectively, partially by regulating the above biomarkers and metabolic pathways. Analyzing and verifying the specific biomarkers, further understanding of the therapeutic mechanism and antihypertensive effect of* Uncaria* was acquired. Metabolomics provided a new insight into estimate of the therapeutic effect and dissection of the potential mechanisms of traditional Chinese medicine (TCM) in treating hypertension.

## 1. Introduction

Hypertension, a complex and multifactorial disease, is closely associated with gene, environment, and metabolic factor, also the most vital risk factor for cardiovascular disease and chronic kidney disease [1]. With the development of study, various theories had been proposed on the pathogenesis of hypertension. Previous research had indicated that endothelial dysfunction might be a crucial mechanism in pathogenesis of hypertension [2]. Under the damage of endothelial cells, active molecules such as ATP and 5-HT would be released. Because these molecules inhibited the activities of NO, CGRP, and other relaxing factors, the activities of ET and TXA2 were increased. Finally, these substances would lead to the increasing blood pressure level [3–5]. In another study, hypertension was considered as a neurogenic disease and it is proposed that the pathology of hypertension was significantly associated with multiple factors in neuroendocrine system [6].

In modern medicine, the hypertension had been studied from above perspectives. In the treatment of hypertension, the traditional Chinese medicine (TCM) has formed a unique theoretical system and control methods. Chinese herbal medicines provided rational means for hypertension.* Uncaria* was employed in treating hypertension as a safe and effective therapy due to characteristics of multiple targets, multiple pathways, and fewer side effects [7].* Uncaria, *with Chinese name of Gouteng and the Latin name of* Uncaria rhynchophylla* (Miq.) from Chinese Pharmacopoeia 2015 version, was firstly recorded in the Chinese medical classics “Compendium of Materia Medica.” Rhynchophylline and isorhynchophylline are the mainly bioactive components in* Uncaria* [8, 9]. Our previous studies indicated that* Uncaria* had effects on lowering blood pressure, protecting vascular endothelial cell, and inhibiting vascular smooth muscle proliferation [10, 11]. The antihypertensive mechanism of* Uncaria* is still unclear, although it has been in widespread application. In modern medicine, the holistic and systemic properties of TCM are consistent with the characters of metabolomics. Therefore, metabolomics might be the bridge between TCM and modern medicine. So metabolomics could be employed to perform such a comprehensive investigation.

Metabolomics, as an important branch of systems biology, provides a comprehensive insight into the rule of metabolite changes. The metabolic variations of organism could be able to be systematically explored with the comprehensive analysis of metabolic result [12]. At present, metabolomics has been widely used in animal model validation [13], disease diagnosis [14], drug toxicity evaluation [15], and drug research and development [16]. And it could evaluate and interpret therapeutic effects of some Chinese medicines [17–19]. Metabolomics analysis required high-density information on a wide range of compound classes and chemistries, as well as high precision. Many technologies, including NMR, GC-MS, LC-MS, and CE-MS [20], have been developed to perform metabolomics analysis. Because of its high sensitivity, high precision, low sample size, and good reproducibility, LC-MS became one of the most frequent significant tools in metabolomics researches [21].

In this study, the metabolic profiling in serums of spontaneously hypertensive rats (SHRs) and drug treated SHRs was analyzed by HPLC-MS. Both antihypertensive efficacy and potential mechanism of* Uncaria* were explored in our experiment. The results of this paper provided further evidence to understand the pathology of hypertension and the intervention effects of* Uncaria*.

## 2. Materials and Methods

### 2.1. Chemicals and Reagents

The dried* Uncaria* (collected in Huaihua, Hunan, China) was authenticated as stem and branch with hook from* Uncaria rhynchophylla* (Miq.) by Professor Feng Li (School of Pharmacy, Shandong University of Traditional Chinese Medicine). The content of* Uncaria rhynchophylline* (main active component in* Uncaria*) was 5.117%, which was determined by HPLC-QQQ/MS (Agilent Technologies, USA). More detailed information was displayed in the Supplementary Materials: MRM parameters and MRM chromatograms of four alkaloids were shown in the Figure a and Table a. The linear equation, linear range, correlation coefficient, quantitative limits, and detection limits of four alkaloids in* Uncaria *were described in Table b.  Table c showed the contents of four alkaloids in the* Uncaria* extract used in this experiment. The methodological results of precision, stability, repeatability, and recovery in the determination of* Uncaria* content were also illustrated in the supplementary material. Acetonitrile of HPLC grade was purchased from Merck (Darmstadt, Germany). Formic acid of HPLC grade was obtained from Tedia (Fairfield, OH, USA). Distilled water was produced by Milli-Q Reagent water system (Millipore, MA, USA). Others were of analytical grade.

### 2.2. Preparation of* Uncaria* and Chemical Profile

Powdered* Uncaria* was extracted with 95% ethanol (10 times, for three times) under thermal reflux for 1 hour. The resulting residue was extracted with water (for three times) with the same condition as ethanol. After filtration, the ethanol and water extracts were combined and concentrated under reduced pressure at 60°C. Finally, the concentration of the stock solution of* Uncaria* extract was adjusted to 1.0 g/crude herb/mL and stored at 4°C.

### 2.3. Animals and Groups

All animals were purchased from the Vital River Laboratory Animal Technology Co. Ltd. (Beijing, China), certificate number SCXK (Beijing) 2012-0001. All studies were carried out by following the guidelines of National Research Council of China and approved by Animal Ethics Committee of Shandong University of Traditional Chinese Medicine. A total of 16 male SHRs (age 12 weeks) were divided randomly into model group (*n* = 8) and an* Uncaria*-treated group (*n* = 8). Another 8 Wistar rats were involved as the normal group. All rats were bred under standard animal conditions with regulated temperature (17–25°C), humidity (45–60%), and 12 h/12 h light/dark cycle; they were admitted to free access to food and water during the study period. In the* Uncaria*-treated group, rats were given* Uncaria* (the dose was 10.001 g raw materials/Kg SHRs). The normal group and model group were given equal quantities of physiological saline. In accordance with the principle of the same volume and the different concentration, all animals were given gastric infusion of one time each day for continuous 4 weeks. One rat was died in* Uncaria* group at the end of experiment. Finally, 23 rats were included in the later experimental analysis.

### 2.4. Blood Pressure Measurement

Blood pressure of rats was measured on tail artery by noninvasive blood pressure measurement before administration and after administration for 1 week, 2 weeks, 3 weeks, and 4 weeks. Acquired data was expressed by means ± SD, and the multigroup comparisons were analyzed by one-way analysis of variance (ANOVA) using SPSS 22.0 (SPSS Inc., USA).

### 2.5. Collection and Preparation of Serum Samples

#### 2.5.1. Serum Sample Preparation

Serum was collected from the abdominal aorta and immediately centrifuged at 4,000 rpm for 15 min. The supernatant was transferred into a clean tube and stored at −80°C. Prior to analysis, 300 *μ*L serum was added with 600 *μ*L acetonitrile and vortexed for 1 min to precipitate the proteins. Then the samples were centrifuged at 12,000 rpm for 15 min and transferred to vials for LC/MS analysis.

#### 2.5.2. Quality Control Sample Preparation

An amount of 40 *μ*L of serum from each rat was mixed to serve as a quality control (QC) sample, which was pretreated as described in Section 2.5.1. QC sample was applied for monitoring the repeatability of samples and validating the stability of HPLC-MS during the analysis. As the sequence running, the QC sample was analyzed six times in the beginning and randomly arranged after 6 unknown serum samples.

### 2.6. HPLC-MS Conditions

HPLC-MS analysis was performed on Agilent 6230 HPLC-TOF system (Agilent Technologies, USA). Chromatographic separation was performed on a Halo-C_18_ column (2.1 × 100 mm, 2.7 *μ*m, America Advanced Material Technology Cor.) with a binary solvents system (solvent A: water with 0.1% formic acid; solvent B: acetonitrile with 0.1% formic acid). The gradient elution program for analysis started from 10% B to 60% B at 0–15 min, was held at 60% for 7 min, and changed to 100% B over 22–24 min. The flow rate was 0.35 mL·min^−1^ and the injection volume was 5 *μ*L. The column temperature was set at 25°C.

*MS Conditions. *They are as follows: capillary voltage, 4.0 kV; drying gas flow, 11 L·min^−1^; gas temperature, 350°C; nebulizer pressure, 35 psig; fragmentor voltage, 140 V; skimmer voltage, 60 V. Data was collected in centroid mode from 100 to 1,000 *m*/*z*. Samples were analyzed randomly for unbiased measurement with reference solution (*m*/*z* 121.050873 and 922.009798) as internal standards to ensure accuracy and reproducibility. Two different fragmentor voltages at 210 and 280 V were applied to search the target ion of biomarkers.

### 2.7. Multivariate Data Analysis and Data Processing

The raw data were firstly converted to.cef format by Mass Hunter Qualitative Analysis Software (Agilent Technologies, USA) and imported into Mass Profiler Professional (MPP, Agilent Technologies, USA) to carry on preliminary calibration of retention time, abundance, and mass range. The data from each sample were normalized to the summed total area and all data were input into Mass Hunter secondly to execute regression analysis. Afterwards data were processed by MPP again to carry on filter flags and significance analysis. Finally, a three-dimensional data matrix which consisted of retention time, mass value, and peak intensity was built. Then the data matrix was imported into SIMCA-P (version 11.5, Umetrics) software in “.csv” format for multivariate statistical analysis; PLS-DA and OPLS-DA were performed for pattern recognition. Variable importance projection (VIP) produced by OPLS-DA, ANOVA, and fold change (FC) was applied to discover the contributable-variable for classification. Finally, the variables with VIP values > 1, *p* value < 0.05, and FC value *⩾* 2 were treated as potential biomarkers. The exact mass of potential biomarkers was searched in database such as HMDB (http://www.hmdb.ca), METLIN (https://metlin.scripps.edu), and KEGG (http://www.genome.jp/kegg/) for biomarkers identification.

## 3. Results

### 3.1. Blood Pressure

**Figure 1:**
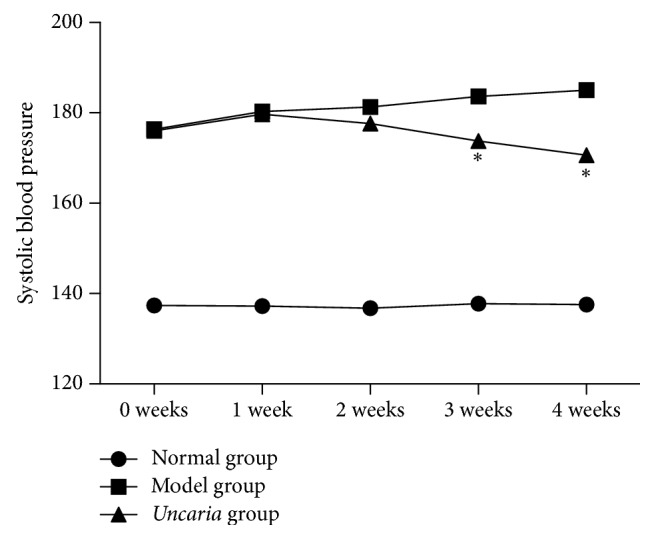
Systolic blood pressure changes in 4-week consecutive administration (mmHg).* Note*. ^*∗*^*p* < 0.05 compared with model group.

Compared with the normal group, the systolic blood pressure (SBP) in the model group was significantly elevated and maintained stable during the drug intervention. Meanwhile, with the treatment of* Uncaria* for 3 weeks, the SBP in* Uncaria*-treated group was lower than model group (*p* < 0.05). With the prolonging of administration time, SBP of* Uncaria*-treated group declined continuously and reached a minimum at the end of the 4th week. These data demonstrated that* Uncaria* had an obvious effect on decreasing blood pressure. The results are shown in Figure 1.

### 3.2. HPLC-MS Analysis of Serum

**Figure 2:**
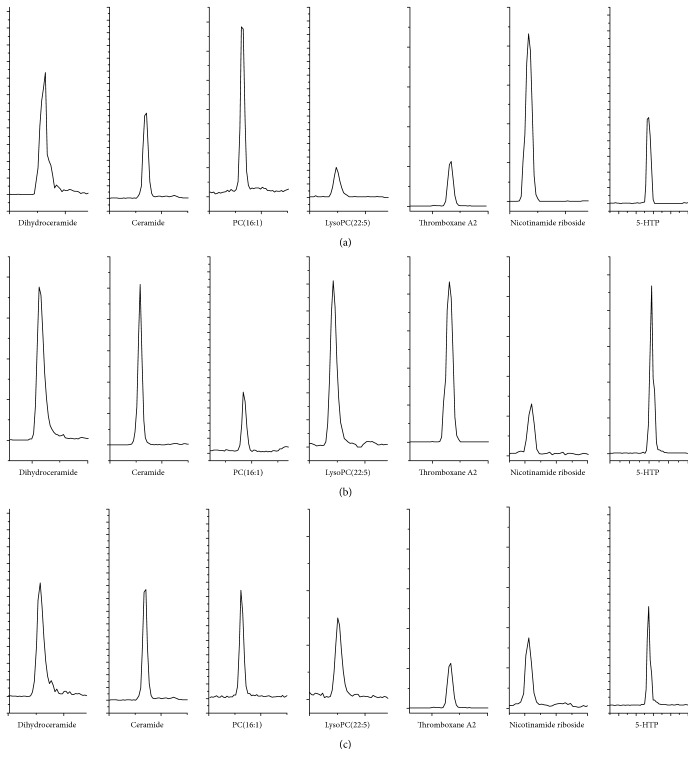
HPLC-MS extract ion chromatogram (EIC) of serum samples from the normal group (a), the model group (b), and the* Uncaria*-treated group (c).

Different kinds of endogenous metabolites could be simultaneously detected with HPLC-MS under the optimized conditions. The extract ion chromatogram (EIC) of serum samples of seven biomarkers from Wistars, SHRs, and* Uncaria-*treated SHRs were presented in Figure 2. It showed the differences of EIC in samples from normal group, model group, and* Uncaria-*treated group.

### 3.3. Data Quality Evaluation

The experimental data of QC samples were imported into SIMCA-P for principal component analysis (PCA). The results showed that the clustering of QC data was closed and concentrated in the 95% confidence interval. This indicated that the data quality was reliable, but the results were not displayed in this paper. In order to validate the reliability of data quality further, 10 ions (the ion's quality number from low to high) were selected from QC samples with the retention time and *m*/*z* pairs of 0.74–113.0909, 0.65–176.0316, 0.64–250.1682, 0.57–363.9451, 17.40–426.3592, 21.51–551.3353, 27.34–676.4716, 8.65–736.1158, 21.54–850.5669, and 17.97–991.6764. The RSDs of peak area were within the range of 0–0.22%, 0–0.60%, and 0–0.50% in three groups; this indicated that the data quality is reliable.

### 3.4. Multivariate Data Analysis

**Figure 3:**
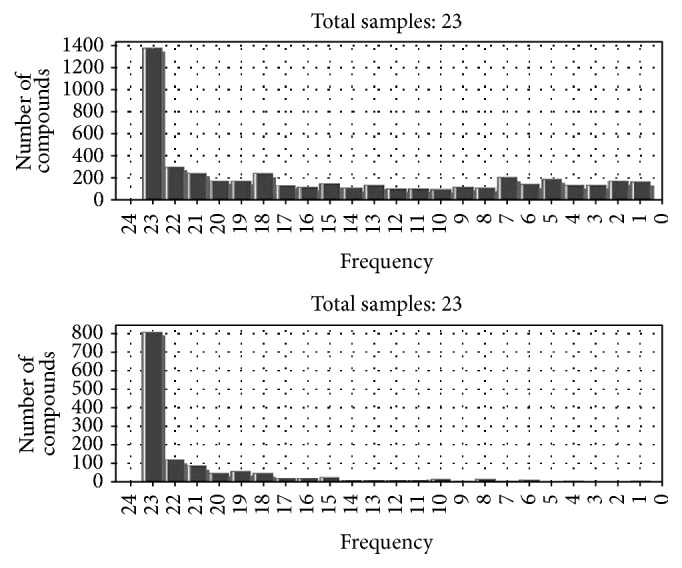
The comparison of results before and after ion regression analysis.

In this study, ion regression was applied to improve the accuracy of the MFE lookup. A calibrated and filtered “.cef” file was input into Mass Hunter secondly to executing regression analysis. The comparison of results before and after ion regression showed that compounds that appeared only 1 time would be significantly reduced after regression, and most compounds appeared in all 23 samples (Figure 3). The results indicated that both the feature search and calibration parameters were successful.

**Figure 4:**
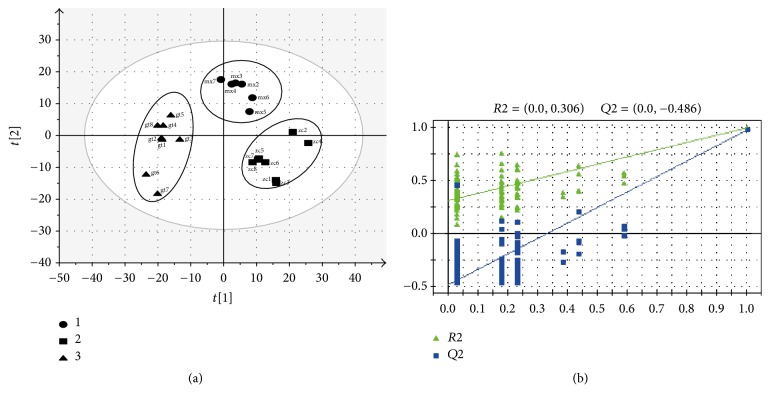
PLS-DA score plot (a) (1: model group; 2: normal group; 3:* Uncaria* group) and validated model plots (b) based on the HPLC-MS data.

PLS-DA is a supervised method for pattern recognition. In PLS-DA score plot (see Figure 4(a)), there was a clear separation in space between the normal and model group, as well as between* Uncaria*-treated group and model group. The results implied that the serum metabolic patterns were changed between the three groups. The* Uncaria*-treated group was much closer to the normal group, which indicated that* Uncaria* did have a potential antihypertensive effect on the SHRs. In addition, the PLS-DA model also enjoyed a good predictive ability and credibility: *R*2*Y* = 0.98; *Q*2 (cum) = 0.83. No overfitting was observed according to the results of permutation test. As shown in Figure 4(b), the *R*^2^*Y*-intercept was 0.306 and *Q*^2^ was −0.486 in the positive ion modes, respectively. Furthermore, all green *R*2-values to the left were lower than the original points to the right, indicating that the original model was valid.

### 3.5. Potential Biomarkers Discovery and Identification

**Figure 5:**
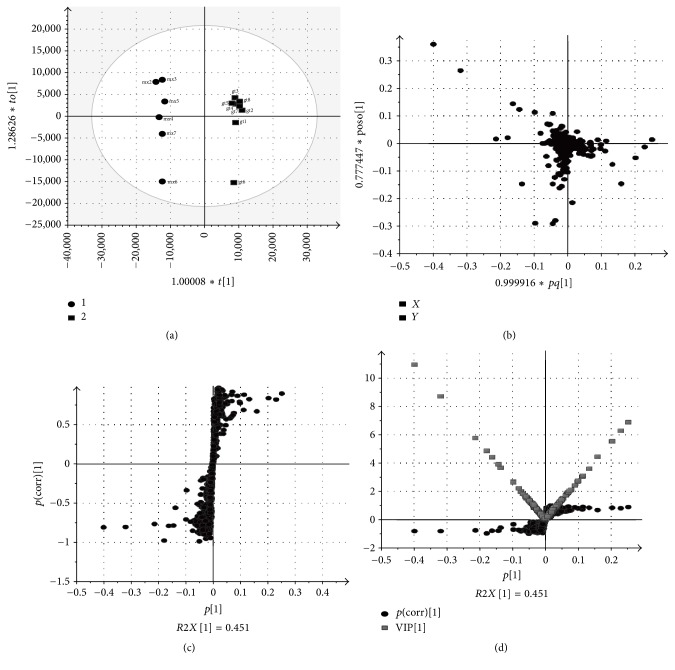
Multivariate analysis of untargeted metabolomics data. (a) OPLS-DA score plots of serum metabolic profiling of model group (1) and* Uncaria* group (2). (b) Loading plots constructed from the supervised OPLS-DA. (c) S-plot constructed from the supervised OPLS-DA. (d) VIP-score plots constructed from the supervised OPLS-DA. The loading plot, S-plot, and VIP plot were carried out to select distinct variables as potential biomarkers.

**Figure 6:**
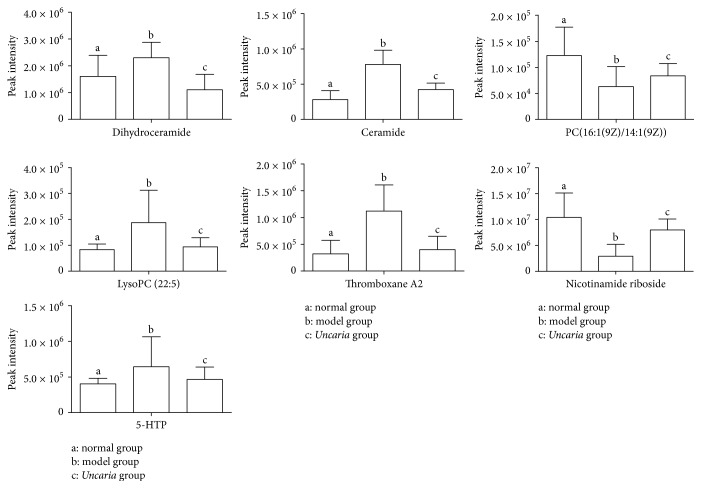
Comparison of different potential biomarkers of normal group, model group, and* Uncaria*-treated group. *p* < 0.05 compared to model group.

The OPLS-DA was utilized to study the blood samples of the* Uncaria* group and the model group. The model evaluation index of *R*^2^*Y* and *Q*^2^*Y* was 0.994 and 0.924, respectively, which indicated that the model had good prediction characteristics. As shown in the OPLS-DA score plot (Figure 5(a)), the* Uncaria* group and the model group can be clearly distinguished. More subtle changes can be found by the loading plot of OPLS-DA (Figure 5(b)), which exhibits each of these variables and is responsible for the separation more intuitively. For further analysis of feature ions, the S-plot (Figure 5(c)) and VIP-plot (Figure 5(d)) from the OPLS-DA were carried out to select distinct variables as potential biomarkers. From the corresponding S-plot and VIP-plot, the ions furthest away from the origin may be regarded as potential biomarkers in* Uncaria* rats. We generated VIP plots from the OPLS-DA with a threshold of 1 to identify the metabolites that significantly contribute to the clustering between groups. Ultimately, 7 potential biomarkers were identified. By comparison with the normal group, 5 metabolites were increased in the model group, and the 2 metabolites were decreased. 7 metabolites tended to normal levels after intervention by* Uncaria*. The differences and change trend of these biomarkers are summarized in Table 1 and Figure 6.

## 4. Discussion

With the deepening research on the pathogenesis of hypertension, a variety of theories have been put forward. At present, the majority of studies suggest that vascular endothelial dysfunction is an important mechanism in the pathogenesis of hypertension [22]. The NO, CGRP, and other diastolic factors' activity declined and ET, TXA2, and other vasoconstrictor substances' secretion increased due to the vascular endothelial dysfunction, which can result in elevating blood pressure [23, 24]. Therefore, the improvement of vascular endothelial function has important significance for the treatment of hypertension. At present, vascular endothelial function becomes a new target for antihypertensive therapy of TCM [25]. Previous studies illustrated that* Uncaria *extract can protect the structure and function of vascular endothelium by reducing vasoconstrictor release, increasing the biological activity of NO, and other ways. These ways can produce vasodilation effects; thereby, the blood pressure was reduced.

**Figure 7:**
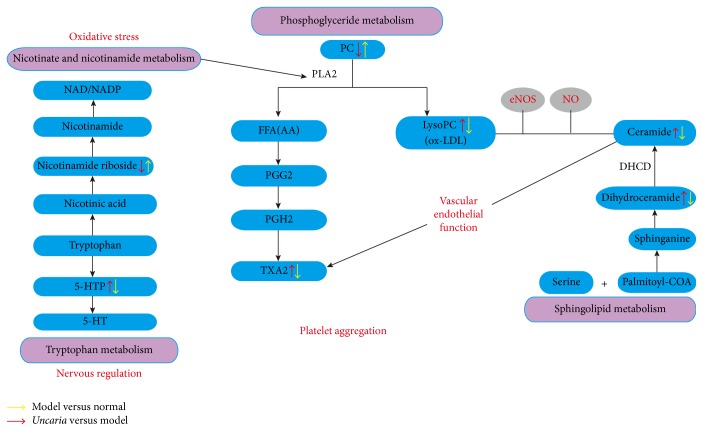
The network of the potential biomarkers variation for SHRs with or without* Uncaria* modulation.

In this paper, a metabolomics study was conducted to analyze the changes of endogenous responses in SHRs after intervention with* Uncaria*. The results suggested that disturbed metabolic profiling was restored in normal level after the treatment of* Uncaria*. 7 biomarkers were identified, and their changes were associated with the hypotensive effect. The biomarkers were mainly involved in three biological pathways: lipid metabolism pathway, amino acid metabolism pathway, and nicotinic acid and nicotinamide metabolism pathway. The above biomarkers and corresponding pathways are associated with vascular endothelial dysfunction and other etiologies of hypertension. At the same time, we proposed a pathogenesis of hypertension associated with metabolic disturbance (Figure 7).

The substance in blue boxes represented biomarkers determined by HPLC-MS, and the pink boxes represented the pathways related to the hypertension.

### 4.1. Lipid Metabolism

The experimental result has revealed that the lipid metabolism disordered, which was consistent with previous conclusions [26]. There is widespread evidence that altering lipid metabolism is the potential pathogenesis of hypertension. Many studies have found abnormal phospholipid metabolism plays an essential role in development of intimal injury and vascular disease. Dyslipidemia may be relevant to the ox-LDL pathway, leading to a decrease of NO and PGI2, as well as the increase of ET-1 and TXA2; both of these effects bring a rise in blood pressure corporately.

A finding showed that sphingolipids were involved in the pathophysiology of hypertension in vivo [27]. Experimental study proved that PKC, Rac, Jun N-terminal kinase, and other protein kinases may be activated by ceramide; then cascade signaling pathways may be initiated to induce cell apoptosis [28]. Thereby, the activation of eNOS was inhibited, oxidative stress response of vascular endothelial cells was induced, and production of NO was decreased, finally leading to vascular endothelial dysfunction [29]. In SHR, the increase of TXA2 release which was induced by ceramide could be caused vasoconstriction, and the vascular constriction was also associated with increased expression of TXA2 synthase [30].

In our study, compared with the model group, dihydroceramide and ceramide were significantly decreased in* Uncaria*-treated group. Their changes were consistent with the antihypertension effect, and the interference of sphingolipid metabolism can be recovered after the administration of* Uncaria*.

By comparison with normal group, the level of TXA2 was upregulated in model group (Figure 6). TXA2 was produced by arachidonic acid in cyclooxygenase pathway, which was able to promote platelet aggregation and vascular contraction [31]. In the pathological process of hypertension, subendothelial collagen and fiber were exposed because vascular endothelium was damaged, combined with platelet to activate and release TXA2. It can increase the peripheral resistance by inhibiting the synthesis of PCI2, affecting the permeability of vascular wall, and promoting the proliferation of smooth muscle cells. Then the blood pressure would be persistently elevated [32, 33]. Furthermore,* Uncaria *made effects on antihypertension through reducing TXA2 level. This result was consistent with the previous study of XIE [34]. Overall, aforementioned variations of lipids suggested lipid metabolism would be disturbed by hypertension, which could be partly regulated and repaired with* Uncaria* treatment.

Phosphoglyceride is one of the most abundant phospholipids in both animal and human body. It is the main component of biofilm, which participates in the signal recognition and transduction in cell membrane. Due to various substituent and replacement groups, phosphoglyceride could be divided into different classes, like phosphatidylcholine (PC), phosphatidylethanolamine (PE), and so on. PC was hydrolyzed by PLA2 from the sn-2 position, producing LysoPC and FFA. The generated endogenous LysoPC was the major active ingredient of oxidized low density lipoprotein (ox-LDL) [3]. LOX-1 receptor could be activated by ox-LDL and lead to dephosphorylation of serine residues in 1179/1177 site of eNOS. Then the activity of endothelial cell eNOS would be inhibited, and endothelium dependent relaxing function was suppressed. All of the above would lead to elevated blood pressure [35]. This confirmed that ox-LDL was an independent risk factor of hypertension. Besides, PLA2 was probably activated by free radicals [36, 37]. In our study, PC was significantly decreased in the model group compared with that in the normal group, and LysoPC does the contrary (Figure 6). There is a tendency to return to normal after intervention by* Uncaria.* The results effectively indicated that* Uncaria* could correct phospholipid metabolism disorder to lower the blood pressure.

### 4.2. Vitamin and Amino Acids Metabolism

In addition to lipid metabolism disordered, the experiment results also find elevated level of nicotinamide ribose and reduced level of 5-HTP. Nicotinamide ribose, which is a component of NADH and NADPH, is precursor of nicotinamide. It exerts protective effects against the oxidative stress process. With the increased level of NADPH, oxidase of generated ROS can be regulated, as well as maintaining the activity of antioxidant enzymes. Thus, the function of endothelial cells can be protected [38, 39]. Furthermore, the activity of PLA2 could be inhibited by NADP to reduce the production of FFA and ceramide [40]. In present research, the variation of 5-HTP indicated that morbidity of hypertension may also be related to tryptophan metabolism. 5-HTP was produced from tryptophan by tryptophan hydroxylase and then transformed to decarboxylate 5-HT. Decarboxylate 5-HT is a potential vasoconstrictor and neuromodulator, which has strong effects in vessel contraction. As a result, 5-HTP was significantly decreased in the* Uncaria*-treated group in comparison with the model group (Figure 6). The reasons for above results may be as follows: firstly, the secretion of neurotransmitters such as norepinephrine could be reduced after* Uncaria *administration, which would inhibit excitation of central nervous system by decreasing level of 5-HT [41]. Secondly, as source of nicotinic acid and nicotinamide, 5-HTP's activity of relevant molecules would be affected by tryptophan. Thus* Uncaria* may play a role in antihypertension through various pathways.

## 5. Conclusion

The serum metabolic profiling was detected and metabolomics was employed to demonstrate the intervention effects of* Uncaria*. In this paper, we observed a clear metabolic characteristic difference between the model group and* Uncaria*-treated groups. The level of 7 perturbed metabolites was reversed. The results suggested that the antihypertensive mechanism of* Uncaria *may involve regulating of lipid, vitamin, and amino acid metabolism, as well as being associated with vascular endothelial function. The results also indicated that* Uncaria* could be employed as a novel and effective antihypertensive treatment herb. In conclusion, metabolomics could give a holistic view of the disease pathogenesis and therapeutics; at the same time it might make great contributions to the evaluation of curative effects of TCM on hypertension.

## Figures and Tables

**Table 1:** Potential biomarkers in samples and corresponding metabolic pathways.

Number	RT	Mass	Biomarker identification	KEGG	Change trend		Pathway
					Trend^a^	Trend^b^	
(1)	15.64	329.3297	Dihydroceramide	C12126	↑	↓	Sphingolipid metabolism
(2)	28.35	563.441	Ceramide	C00195	↑	↓	Sphingolipid metabolism
(3)	29.94	701.2065	PC(16:1(9Z)/14:1(9Z))	C00416	↓	↑	Glycerophospholipid metabolism
(4)	18.39	569.3487	LysoPC (22:5)	C04230	↑	↓	Glycerophospholipid metabolism
(5)	26.68	296.2828	Thromboxane A2	C02198	↑	↓	Arachidonic acid metabolism
(6)	26.68	255.2564	Nicotinamide riboside	C03150	↓	↑	Nicotinate and nicotinamide metabolism
(7)	0.68	220.1793	5-HTP	C00643	↑	↓	Tryptophan metabolism

*Note*. ^a^Trends of model group compared with normal group of metabolites. *p* < 0.05 compared to normal group. ^b^Trends of *Uncaria* group compared with model group of metabolites. *p* < 0.05 compared to model group. ↑: upregulated. ↓: downregulated.

## Supplementary Materials

Supplementary Materials
* Uncaria* could be found in Chinese Pharmacopoeia 2015 version, but no active compounds were determined. According to the related reference, we determined four alkaloids including rhynchophylline, isorhynchophylline, corynoxeine, and isocorynoxeine by HPLC-QQQ-MS. Because the topic of the manuscript is metabolomics, it is shown here. (1) HPLC-QQQ-MS/MS instrumentation and analytical conditions: Table a: parameters of LC-QQQ/MS/MS analysis for four constituents (alkaloids). Figure a: MRM chromatograms of four alkaloids in* Uncaria* extracts (chromatography of* Uncaria* extract). (2) Methodology: the precision, stability, repeatability, and recovery were also measured. Table b: linear equation, linear range, correlation coefficient, and detection limits of four constituents (alkaloids). Table c: the content of four constituents (alkaloids) in* Uncaria* extract.
