# Supplementary material for: Serum Metabolomics Study Based on LC-MS and Antihypertensive Effect of Uncaria on Spontaneously Hypertensive Rats
Source: Evid Based Complement Alternat Med. 2018 Apr 4;2018:9281946. doi: 10.1155/2018/9281946 (PMC5904782; doi:10.1155/2018/9281946)
Supplement: Supplementary Materials — Uncaria could be found in Chinese Pharmacopoeia 2015 version, but no active compounds were determined. According to the related reference, we determined four alkaloids including rhynchophylline, isorhynchophylline, corynoxeine, and isocorynoxeine by HPLC-QQQ-MS. Because the topic of the manuscript is metabolomics, it is shown here. (1) HPLC-QQQ-MS/MS instrumentation and analytical conditions: Table a: parameters of LC-QQQ/MS/MS analysis for four constituents (alkaloids). Figure a: MRM chromatograms of four alkaloids in Uncaria extracts (chromatography of Uncaria extract). (2) Methodology: the precision, stability, repeatability, and recovery were also measured. Table b: linear equation, linear range, correlation coefficient, and detection limits of four constituents (alkaloids). Table c: the content of four constituents (alkaloids) in Uncaria extract. [file 9281946.f1.doc]

As a well known medicinal plant, while Uncaria could found in Chinese Pharmacopoeia 2015 version, but not active compounds were determined. According to the related reference, we determined four alkaloids including rynchophylline, isorhynchophylline, corynoxeine and isocorynoxeine by HPLC-QQQ-MS. Because the topic of manuscripts is metabolomics, so it was shown in supplementary and as follows:

*HPLC-QQQ-MS/MS instrumentation and analytical conditions*

In the quantitative analysis, An Agilent 6420A triple quadrupole LC-MS system (Agilent Corporation, MA, USA) equipped with G1311A quaternary pump, G1322A vacuum degasser, G1329A autosampler and G1316A therm was employed. The chromatographic condition was the same with that for qualitative analysis. Nitrogen was used as nebulizer gas. The MS conditions were optimized as follows: spray voltage: 4000 V for positive mode; Nebulizer pressure: 35 psi; Gas temperature: 350 ℃ and delivered at a flow rate of 10 L^.^min^-1^. The most appropriate fragmentor energies and collision energies used for four constituents were adjusted according to each analyte respectively , the optimized condition and the representative extract ions were listed in Table a. The peak widths of precursor and product ions were maintained at 0.7 amu at half-height in MRM mode. The MRM chromatograms of four alkaloids were shown in Figure a.

TABLE a: MRM Parameters of LC-QQQ/MS/MS analysis for four alkaloids

| No.(RT) | compounds | Fragmentor | Precursor Ion  and product ion | Collision Energy | Collision Energy |
| --- | --- | --- | --- | --- | --- |
| 1  (42.3~44.6min) | rynchophylline | 165 V | 385-160 | 35 V | 21 V  27 V |
| 2  (46~49.4min) | corynoxeine | 160 V | 383-160 | 31 V | 18 V  25 V |
| 3  (52.6~55.1min) | isocorynoxeine | 160 V | 383-160 | 31 V | 18 V  25 V |
| 4  (55.2~58.1min) | isorhynchophylline | 165 V | 385-160 | 35 V | 21 V  27 V |


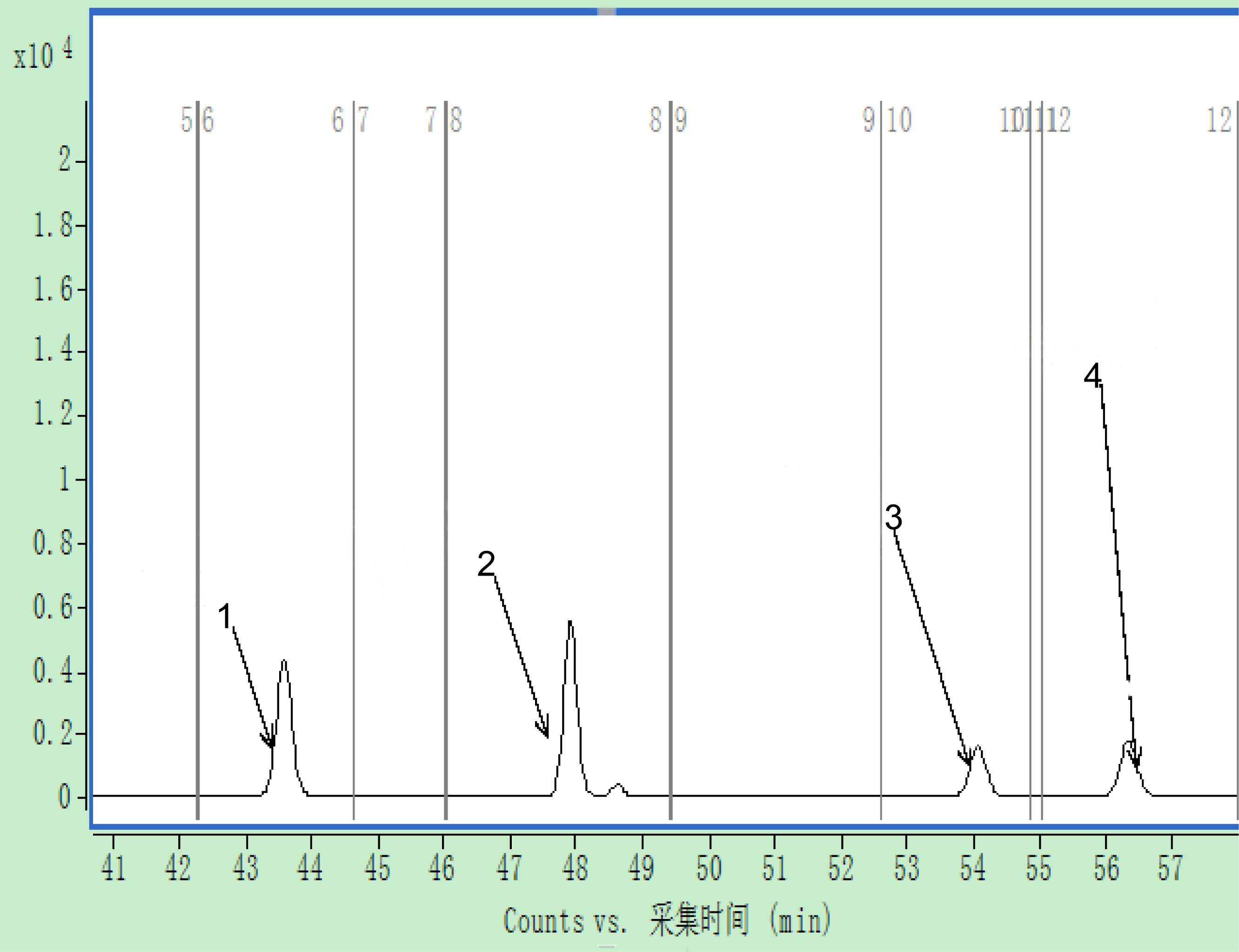


FIGURE a: MRM chromatograms of four alkaloids in *Uncaria* exacts

(1. rynchophylline 2. corynoxeine 3. isorhynchophylline 4. isocorynoxeine )

*Linearity of calibration curves, limit of detection (LOD) and limit of quantification (LOQ)*

The standard solutions were diluted with chromatographic grade methanol to seven different concentrations for the construction of calibration curves. All the calibration curves indicated good linearity with determination coefficients (r) from 0.9960 to 1.0000. The limits of detection (LOD) and the limits of quantification (LOQ) were evaluated at a signal-to-noise ratio (S/N) of 3/1 and 10/1 respectively. The parameters of LOD and LOQ for each constituent in this experiment were shown in Table b.

TABLE b: Linear equation, linear range, correlation coefficient, quantification limits and detection limits of four constituents

| compounds | Regression equations | Correlation coefficients /r | Linear ranges  /μg·L^-1^ | Quantification limits/μg·L^-1^ | Detection  /μg·L^-1^ |
| --- | --- | --- | --- | --- | --- |
| rynchophylline | y=166.61x+9768.47 | 0.9996 | 40.72~10180 | 4.24 | 1.33 |
| isorhynchophylline | y=200.63 x+7295.60 | 0.9998 | 32~8000 | 3.36 | 1.08 |
| corynoxeine | y=271.52x+4237.11 | 0.9999 | 24~6000 | 3.15 | 1.01 |
| isocorynoxeine | y=172.59x+2308.19 | 1.0000 | 17.92~4480 | 4.00 | 1.26 |

*Precision, stability and repeatability*

To evaluate the precision of the present method, the intra-day precisions were calculated by analyzing the standard solution under the optimized experimental conditions, which respective RSD values were 2.61% ~ 4.31%. The stability of each constituent in 24h at room temperature (n=6) were 2.85%~4.58%. Furthermore, sample solutions for *Uncaria* were prepared in parallel (n=6) to evaluate the repeatability and achieved the RSD of 1.01%~1.79%.

*Recovery*

The recovery was used to evaluate the accuracy of the method. Six copies of *Uncaria exact* were taken for recovery test and standard solutions of four constituents were added according to the levels (1:1) respectively. The mixtures were treated as the procedure of sample preparation and analyzed using the method described above. Recovery (R) was calculated as R=100(M_measured_−M_initial_)/M_added_ (M_measured_ = measured amount in the recovery sample, M_initial_ = initial amount in the sample, M_added_ = amount in the standard solution used) for each compound. The average recovery rate of each constituent was 94.6% ~ 96.5%, with the RSD value less than 3%, which can meet the requirements for determination of the contents of four constituents in Uncaria exact.

*Analysis of samples*

This validated HPLC-QQQ/MS/MS method was used for the quantification analysis of four constituents in Uncaria exact in the MRM mode, the MRM chromatograms were shown in Figure a Each constituent was calculated by their respective calibration curve. The content of four constituents in Uncaria exact were listed in Table c.

TABLE c: The content of four constituents in Uncaria exact

| No. | rynchophylline /μg·g^-1^ | isorhynchophylline /μg·g^-1^ | corynoxeine  /μg·g^-1^ | isocorynoxeine /μg·g^-1^ |
| --- | --- | --- | --- | --- |
| sample 1 | 352.4569 | 145.1206 | 94.5679 | 205.3518 |
| sample 2 | 354.1862 | 143.3568 | 93.2486 | 202.8464 |
| sample 3 | 359.9861 | 143.1205 | 92.0168 | 199.7869 |
| average | 355.5431 | 143.8660 | 93.2778 | 202.6617 |
